# Self-Efficacy in High-Performance Sports: A Systematic Review and Meta-Analysis

**DOI:** 10.11621/pir.2025.0107

**Published:** 2025-03-01

**Authors:** Larién López-Rodríguez, Luis G. González Carballido, César A. Montoya-Romero, Marisol C. Suárez-Rodrígueza, Melvys González-Rabeiro, Osniel Charlot-Cardoza, Abel Yañez-Rivera, Adrián Feria-Madueño

**Affiliations:** a Sports Medicine Institute, Havana, Cuba; b University of Seville, Spain

**Keywords:** self-efficacy, sports, high-performance, meta-analysis, review

## Abstract

**Background:**

Studies of self-efficacy in sports have demonstrated its importance in performance. These have been of English-speaking and Latin American origin, mostly descriptive, qualitative, and relate it essentially to emotional variables; interventions reveal its sources and the way to improve it.

**Objective:**

A meta-analysis and systematic review of self-efficacy studies in high- performance sports in 2015–2022.

**Design:**

The PRISMA method and a flow diagram were used. The databases were SciELO, Dialnet, Redalyc, CORE, REBID, Science Research, Google Scholar, and PubMed. The dimensions of analysis were: descriptions of the articles; methodological approach, and characteristics of the self-efficacy measurements. The keywords “self-efficacy” and “sports,” along with their translations into Spanish, were connected using the OR Boolean operator. The inclusion of “high performance” or “elite” was carefully considered to avoid unintended exclusions.

**Results:**

Thirty-four articles were selected, with soccer and basketball being the most studied sports. Descriptive and correlational designs predominated: increasing relationships between two or more variables with self-efficacy, the studies that discussed interventions were the only ones that addressed longitudinal studies. In the measurements, those of general self-efficacy predominate according to the tasks faced by the athletes; individual self-efficacy is more highly valued; strength and generality are considered in the microanalyses.

**Conclusion:**

Progress has been shown in studies of self-efficacy in high-performance sports. There remain opportunities for longitudinal studies, instruments with sport-specific indicators, analysis of levels and collective self-efficacy that will allow researchers to further explain the phenomenon

## Introduction

In the realm of motivation, any scholarly investigation must possess a grand scope, compelling objectives, and inherent complexity stemming from its multifaceted components (Morris et al., 2022). These attributes, eloquently articulated by Fernández-Abascal (1997), likely account for the polysemous nature of the practices of psychologists and other professionals dedicated to the study and management of human behavior. Therefore, it is imperative to approach this subject matter with rigorous scientific methodologies and theoretical underpinnings.

In the domain of sports, precise terminology becomes even more indispensable, particularly when examining concepts such as self-efficacy. Developed by Bandura (1977), the theory of self-efficacy encapsulates the belief in one’s capacity to successfully execute tasks at varying levels of complexity. This category comprises two intertwined components: outcome expectation, which pertains to the anticipation of specific results from certain actions, often with a professional and impersonal focus, and self-efficacy expectation or personal efficacy expectation, referring to the belief in one’s capability to achieve success through individual actions.

Numerous studies have established the nexus between self-efficacy and motivation (Bandura, 2000; Cartagena, 2018; Girardi et al., 2018; Ornelas et al., 2012), showcasing their susceptibility to emotional influences and performance outcomes, and their role in fostering a sense of security when approaching established standards (Feltz, 2007; Mananis et al., 2020; Montoya et al., 2020). Moreover, research in sports psychology underscores the significant impact of self-efficacy on performance and motor tasks, aiding in the prediction of athletic outcomes (Feltz, 1992; Habeeb et al., 2019; McLean et al., 2020). These investigations span diverse sporting disciplines, employing experimental and observational methodologies, and utilize instruments such as the Physical Self-Efficacy Questionnaire and task-specific scales to assess self-efficacy in sports contexts.

Scholars have delved into the various sources of self-efficacy, encompassing achievement experiences, verbal persuasion, vicarious experiences, and physiological cues (Bandura, 1998; Feltz & Riessinger, 1990; Weinberg et al., 1982), often through experimental inquiries to elucidate their impact on performance outcomes. While descriptive inquiries into self-efficacy in sports have proliferated (Abalde & Pino, 2016; De Andrade et al., 2019), studies grounded in experimental designs and theoretical frameworks related to performance are relatively scarce.

Meta-analyses and reviews focusing on self-efficacy in high-performance sports have predominantly adopted a qualitative lens, highlighting its predictive utility and influence on athlete preparation. Notable scholarly works from English-speaking and Latin American sources have elucidated the dynamics of performance in conjunction with psychological and formal variables, shedding light on fundamental sources and strategies to enhance self-efficacy, particularly in post-injury rehabilitation settings (Balaguer et al., 1995; Brinkman et al., 2019; Guillén, 2007; Moritz et al., 2000; Ol-ortegui, 2020; Yevilao, 2019).

High-performance sports are characterized by rigorous physical and mental demands during training, necessitating the convergence of sophisticated physical, athletic, and psychological techniques. This integration stems from interdisciplinary research on human capabilities aimed at achieving goals and fostering efficacy in task execution, underscoring the pivotal role of psychological intervention (Guillen, 2007, pp. 21–32).

Acknowledging the imperative to advance research on self-efficacy in sports, Yevilao (2019) examined its relationship with motivation, effort, performance, thought control, and attribution of successes and failures.

There is a pressing need to develop specialized assessment tools and novel methodologies to explore self-efficacy in activity-specific contexts and devise intervention programs to further elucidate this construct.

The general objective of this study is to analyze the current state of self-efficacy in high-performance sports and propose future directions for research development in this field. Specifically, it aims to characterize the most relevant publications in terms of geographical and temporal scope, identify the most commonly used study designs in this area, explore the main relationships between self-efficacy and variables such as sports performance and motivation, as well as review the most frequently used instruments to assess self-efficacy and the interventions implemented to enhance it in high-performance sports contexts.

## Methods

This manuscript presents a systematic review conducted in accordance with the PRISMA (Preferred Reporting Items for Systematic Reviews and Meta-Analyses) guidelines, which are recognized standards for reporting systematic literature reviews and meta-analyses. The PRISMA framework, comprising a 27-item checklist and a four-phase flow diagram, aids in the comprehensive reporting of such studies (Page et al., 2021). While primarily intended for randomized trials, PRISMA is adaptable for systematic reviews across various research types, particularly intervention evaluations, thereby facilitating critical assessments of published systematic reviews.

In this review, empirical evidence is presented, offering a scientific approach to identifying and analyzing previous studies (Botella & Sánchez-Meca, 2015) on the subject of self-efficacy in high-performance sports, reported in publications from 2015 to 2022. The decision to include publications from 2015 to 2022 in this systematic review ensures that the most recent and relevant evidence on self-efficacy in high-performance sports is captured. This period was selected for several reasons. First, it reflects advances in knowledge and methodology, as the last decade has witnessed significant developments in sports science, including new measurement techniques and analytical tools that align with current standards and theories. Focusing on this timeframe ensures that the included studies are methodologically robust and consistent with contemporary research practices. Second, prioritizing recent evidence enhances the relevance and applicability of the findings, particularly in a field that evolves rapidly. Earlier studies may rely on outdated methodologies or contain findings that are no longer applicable in the current context. Third, this period coincides with a growing interest in self-efficacy research, capturing key publications and trends that have shaped the field in recent years. Finally, limiting the scope to recent years is a common practice in systematic reviews to ensure a manageable volume of studies while maintaining the validity and applicability of the results. Together, these considerations justify the chosen timeframe and reinforce the review’s contribution to advancing current understanding in the field.

### Search Strategy

The search strategy encompassed three key indicators: databases, keywords, and exclusion criteria. Databases such as SciELO, Dialnet, Redalyc, CORE, REBID, Science Research, Google Scholar, and PubMed were selected based on their international visibility and recognition. The keywords “self-efficacy” and “sport,” along with their translations into Spanish, were connected using the OR Boolean operator (Avelar-Rodríguez & Toro-Monjarez, 2018). The inclusion of “high-performance” or “elite” was carefully considered to avoid unintended exclusions, with specific emphasis placed on high performance in subsequent analyses.

### Study Selection

The filters applied in the databases included publications from 2015 to 2022, full-text articles, and publications in English, Spanish, or Portuguese. The inclusion of English was prioritized as it is the most widely used language in scientific publications, facilitating global dissemination and visibility (Drubin & Kellogg, 2017). Spanish was selected due to its rising prominence in scientific research. Portuguese was included as it represents a major language within Ibero-American scientific production, offering significant opportunities for knowledge dissemination both within the European Union and among the Community of Portuguese Language Countries (Badillo, 2021). Exclusion criteria were applied at multiple levels to ensure the relevance and quality of the selected articles, with duplicate articles being removed initially, followed by exclusion of articles not related to self-efficacy in high-performance sports and those not published in journals indexed in SJR and/ or JCR. Review articles were further excluded, and the remaining information was compiled for subsequent analysis.

### Evaluation of Risk of Bias and Quality of Selected Articles

Two approaches were employed to assess the quality of the selected articles. The first approach utilized evaluation criteria established by previous authors, including Barba et al. (2020), López & Gené-Morales (2021), and Garcés de Los Fayos et al. (2023). The second approach was based on theoretical dimensions guiding the analysis process. Three authors with extensive research experience evaluated the quality of the articles based on predefined criteria, including description of methodology, inclusion in JCR/SJR, sample size, and duration. Total scores were calculated for each article and classified as low quality (LQS), moderate quality (MQS), or high quality (HQS), based on predefined thresholds. The criteria were as follows:

Description of the methodology (“0”: not included, “1”: brief description, “2”: detailed description); inclusion in JCR/SJR, publication of the study in a journal indexed in JCR and/or SJR (“0”: not indexed, “1”: indexed in SJR, “2”: indexed in JCR); sample size (“0”: less than 10 participants, “1”: 10 to 50 participants, “2”: more than 50 participants); duration (“0”: less than eight sessions, “1”: 9 to 14 sessions, “2”: more than 15 sessions); JCR, Journal Citation Reports; SJR, Scimago Journal Rank; HQS (7–10): high-quality study; MQS (4–6): moderate-quality study; LQS (0–3): low-quality study.

After evaluating the articles, total scores were calculated for each one, and they were classified into three categories based on quantitative criteria. Articles scoring below 3 points were classified as LQS, those scoring between 4 and 6 points as MQS, and those scoring above 7 points as HQS. These thresholds were established following the methodologies and guidelines proposed by Barba et al. (2020), López & Gené-Morales (2021), and Garcés de Los Fayos et al. (2023).

#### Analysis Dimensions of Selected Articles for Synthesis

1) Descriptive analysis of formal characteristics of the selected articles included calculation of frequency of journal titles, language, country of research, sports disciplines studied, and distribution of publications across the analyzed years (2015–2022).
2) Methodological procedures of the articles were analyzed, including the percentage distribution of research designs adopted, differentiation between descriptive, correlational, explanatory, and validation studies (Hernández-Sampieri & Mendoza, 2018). Correlational and explanatory studies identified variables or categories associated with self-efficacy, while experimental designs analyzed self-efficacy as both an independent and dependent variable. Various sources of self-efficacy were explored, along with validations of measurement instruments.
3) Self-efficacy measurements were examined for general and specific evaluations, considering Bandura’s dimensions for microanalysis. Additionally, studies evaluating self-efficacy individually and collectively were included, with collective self-efficacy defined as a shared sense of collective competence.

Quantitative analysis of the collected information was performed using IBM SPSS Statistics for Windows version 25.0 (IBM Corp. Released, 2017).

This systematic review provides a comprehensive synthesis of empirical evidence on self-efficacy in high-performance sports, offering valuable insights into methodological approaches, quality assessment, and analysis dimensions of the selected articles.

## Results

**Figure 1:**
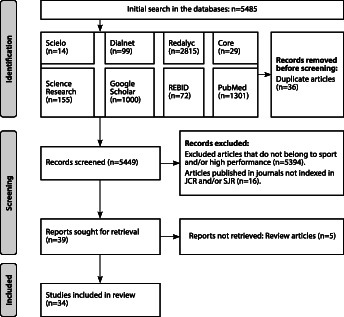
Flowchart for Article Selection

The search and selection process yielded a total of 34 articles meeting the inclusion criteria, following a systematic screening and evaluation procedure (*Figure 1*).

These articles offer valuable insights into the multifaceted dimensions of self-efficacy in high-performance sports, spanning diverse sports disciplines and methodological approaches (*Table 1*).

**Table 1:** Coding of the Studies Based on the Author, Year of Publication, Journals, Study Objective, Participants, Sport, and Country

Authors	Year	Journals	Study objective	N	Sport	Country
Leo, González-Ponce & Sánchez	2015	Journal of Sport Psychology	Simultaneously analyze the empirical relationship between two variables posited as antecedents, role conflict, and team conflict (task and relationship), and the collective efficacy of professional female soccer players.	225	Soccer	Spain
Rivas, Ponzanelli, López de la Llave, Pérez & Garcia-mas	2015	Revista Mexicana de Psicología	Examine the levels of collective efficacy related to the required behaviors for effective performance as competitive soccer players and compare it with individuality-collectivity regarding the willingness to work in a team.	112	Soccer	Mexico
Rodríguez, López, Gómez, Rodríguez & Granada	2015	Revista Iberoamericana de Psicología del Ejercicio y el Deporte	Verify, through a case study, the effect of a training program on activation control using breathing techniques and biofeedback on self-efficacy and putting effectiveness.	4	Golf	Colombia
Argudo, Ricardo de la Vega & Ruiz	2015	Apunts. Educación Física y Deportes.	Analyze the degree of consistency between a high-level water polo goalkeeper’s perception of behavioral success and their observable performance in competition.	1	Water polo	Spain
Gacek	2015	Roczniki Panstwowego Zakladu Higieny	Investigate dietary behaviors and their association with general self-efficacy.	100	Football	Poland
Estevan, Álvarez & Castillo	2016	Cuadernos de Psicología del deporte	Develop the Perceived Self-Efficacy Questionnaire in Taekwondo Actions (CAPAT) and analyze its psychometric properties; 2) Examine differences in the perception of technical-tactical self-efficacy among taekwondo practitioners based on gender and combat success; and 3) Analyze the technical-tactical performance based on gender and combat success of the athlete. Construction and validation of a self-confidence scale for both individual and team sports in competitive situations.	85	Taekwondo	Spain
Martínez-Romero, Molina & Oriol- Granado	2016	Cuadernos de Psicología del deporte.	Construction and validation of a self-confidence scale for both individual and team sports in competitive situations.	307	Soccer, boating basketball, cycling, roller hockey, tennis, volleyball, handball	Chile
Abalde & Pino	2016	Retos	Examine whether individuals with higher perceived self-efficacy for sports and higher self-esteem achieve better sports outcomes.	56	judo	Spain
Tejero-González, De la Vega-Marcos, Vaquero-Maestre & Ruiz	2016	Journal of Sport Psychology	1) Describe the levels of life satisfaction and mobility self-efficacy among individuals with physical disabilities who practice wheelchair basketball, 2) Contrast these levels based on the participants’ level of competition or sports excellence, and (3) Measure the association between the life satisfaction of these individuals and their age, functional classification, and wheelchair mobility self-efficacy perception.	103	Wheelchair basketball	Spain
Gacek	2016	Roczniki Panstwowego Zakladu Higieny	Analyze the association between the level of general self-efficacy and the use of dietary supplements in competitive Polish athletes playing American football.	100	Football	Poland
García- Naveira	2017	Deportes CCD (Cultura, ciencias y deportes)	Understand optimism, competitiveness, and self-efficacy, their relationship, and differences based on gender.	72	Athletics	Spain
Peinado, Cocca, Solano & Blanco	2017	Journal of Sport Psychology	Evaluate the factorial structure of the Self-Efficacy Scale in Food and Physical Health Care (ECASF) and verify its psychometric equivalence in athletes and non-athletes.	637 athletes and 668 nonathletes	College selective teams. Does not specify.	Mexico
Segura, Adanis, Barrantes- Brais, Ureña & Sánchez	2018	MH Salud	Analyze the relationships between self-efficacy, pre-competitive anxiety, and subjective perception of sports performance.	32	Soccer	Costa Rica
García-Naveira	2018	Cuadernos de Psicología del Deporte	Examine the relationship between specific self-efficacy (perceived competence in defense and attack) and individual sports performance (objective and subjective measures of performance in attack and defense).	172	Soccer	Spain
Molina, Granado & Mendoza	2018	Journal of Sport Psychology	Investigate the influence of athlete’s autonomy support relationships on their positive effectiveness, emotional regulation experienced in competition, and the acquisition of personal resources such as self-confidence and self-efficacy.	300	Boating, swimming, tennis, taekwondo, athletics, rhythmic gymnastics, volleyball, basketball rugby	Chile
Rubio, Her nán dez, Sánchez-Iglesias, Cano & Bureo	2018	Journal of Sport Psychology	1) Compare athletes’ self-efficacy beliefs upon arrival at the facilities and immediately after receiving the coach’s pep talk.. 2) Analyze the influence of pre-game talks in eight matches following the same procedure with a sample of ten male players from the same team.	61	soccer	Spain
Malinaukas, Sniras & Malinaukienes	2018	Journal of Sport Psychology	Evaluate the effectiveness of an educational program aimed at improving social self-efficacy among students who practice basketball.	54	Basketball	Lithuania
Olmedilla, Rubio, Fuster-Parra, Pujals & García-Mas	2018	Frontiers in Psychology	Construct a Bayesian network to assess probabilistic links between relevant psychological variables and the occurrence of injuries.	297	Athletics, swimming kayak, rowing, figure skating, judo, fencing, boxing, karate, basketball, handball, soccer.	Spain
De Andrade, Gattás & Moura	2019	Revista Brasileira de Medicina do Esporte	Examine the validity of the Individual Zone of Optimal Functioning (IZOF) model from a multidimensional anxiety perspective and investigate the possibility of expanding the IZOF theory to the construct of self-efficacy.	7	volleyball	Brazil
Salles, Soares, Collet, Milan, Palheta, Mendes, Kós, Nascimento & Carvalho	2019	Cuadernos de Psicología del Deporte	Analyze the variation of collective efficacy among young basketball players, considering the influence of chronological age and maturational stage.	57	Basketball	Brazil
Chen, Zhang, Yin, Li, Cao, Gutiérrez- García & Guo	2019	Frontiers in Psychology	Explore the relationship between self-efficacy and aggressive behavior, as well as the mediating effect of self-control.	414	Boxing	China
Walter, Nikoleizig & Alfermann	2019	Sports	Verify the impact of self-talk on psychological and performance outcomes (competitive anxiety, volitional skills, selfefficacy, and performance ratings).	117	Boating, artistic gymnastics, rhythmic gymnastics, judo, swimming, wrestling, ice hockey, handball, volleyball	Germany
Reigal, Vázquez- Diz, Morillo-Baro, Hernández-Mendo & Morales-Sánchez	2019	International Journal of Environmental research and public health	1) Analyze the relationships between sport psychological profile, competitive anxiety, mood, and self-efficacy in Beach Handball players. 2) Determine the predictive capacity of the psychological profile on competitive anxiety, mood, and selfefficacy.	181	beach handball	Spain
Martínez-Alvarado, García, Palacios & Rodríguez	2020	Revista Iberoamericana de Diagnóstico y Evaluación e Avaliação Psicológica	Analyze the psychometric properties of the Collective Efficacy Questionnaire for Sports (CEQS).	935	Soccer, football soccer flags, basketball	Mexico
Chen, Qiu, Chen, C., Qang, Zhang, & Chai	2020	Frontiers in Psychology	Explore the relationship between boxers’ self-efficacy and depression, as well as the mediating effect of self-control.	231	Boxing	China
Carreres- Ponsoda, Escartí, Jimenez-Olmedo & Cortell-Tormo	2021	Frontiers in Psychology	Implement the Teaching Personal and Social Responsibility (TPSR) model in a competitive context and analyze differences between the intervention group and the control group.	34	Soccer	Portugal
Monteiro, D. Monteiro, M. Torregrossa & Travassos	2021	Frontiers in Psychology	Examine the role of self-efficacy, professional goals, and athletic identity in the career planning of elite soccer players, proposing a model.	281	Soccer	Portugal
Goraczko, Zurek, Lachowicz, Kujawa & Zurek	2021	International Journal of Environmental research and public health	Investigate self-efficacy, quality of life, and their correlations among outstanding athletes who have experienced spinal cord injuries and determine if these individuals have specific psychological characteristics contributing to better quality of life.	32	Wheelchair dance, car racing, rugby, wheelchair basketball, canoe, skiing, hand cycling	USA, United Kingdom, Canada, Brazil, Poland, Austria, Australia, Japan, South Africa.
Ramolale, Malete & Ju	2021	Frontiers in Psychology	Examine whether mental toughness mediates the relationship between self-efficacy and prosocial/antisocial behaviors in young athletes from Botswana.	158	Soccer, netball, athletics, volleyball, softball, boxing	Botswana
Peng & Zhang	2021	Frontiers in Psychology	Examine the moderating effects of goal orientations and self-efficacy on competitive cognitive anxiety and motor performance in conditions with varying levels of ego threats.	81	basketball	China
Stankovic, Todorovic, Miloševic, Mitrovic & Stojiljkovic	2022	Frontiers in Psychology	Compare trained athletes and athletes from various team sports in terms of aggression manifestation, personality traits, emotional intelligence, and aggression self-efficacy.	140	Judo, handball, soccer, water polo	Serbia
Tang, Liu, Jing, Wang & Yang	2022	International Journal of Environmental Research and Public Health	Verify the effects of mindfulness interventions on competitive anxiety and burnout in injured athletes returning to sports.	433	Does not specify.	China
Kwon, Shin & Shin, M	2022	International Journal of Environmental Research and Public Health	1) Verify the validity and reliability of the Korean versions of the Observational Learning Function Questionnaire and the Self-Efficacy Questionnaire. 2) Examine whether observational learning predicts self-efficacy under different pressure and personal performance conditions. 3) Test the doubly mediating effect of observational learning and the effect of winning or losing attributions on self-efficacy.	211	Basketball, ice hockey, baseball, archery, tennis, judo, soccer, rugby	Korea
Rogowska, Tataruch, Niedz´wiecki & Wojciechowska- Maszkowska,	2022	International Journal of Environmental Research and Public Health	Explore the relationship between motivational system focus, self-efficacy, and athletic success among athletes, while controlling for gender, sports discipline, and athletic level.	156 (54-102)	Speed skating, athletes studying physical education (does not specify).	Poland

Risk of bias and quality assessment of the selected articles were evaluated. Of the 34 articles included in the review, 3 were classified as high-quality studies (HQS), 31 as medium-quality studies (MQS), and none as low-quality studies (LQS). Among these studies, 22.5 of HQS were indexed in JCR and SJR; 73.5 of the articles were cross-sectional, indicating that the duration criterion significantly influenced the total scores. The evaluation of the quality of the articles can be found in *Table 2*.

**Table 2:** Evaluation of the Quality of the Articles

Studies	Methodology	Inclusion in JCR/SJR	Sample	Time	Total score	Quality level
Leo, González-Ponce & Sánchez (2015)	2	2	2	0	6	MQS
Rivas, Ponzanelli, López de la Llave, Pérez &Garcia-mas (2015)	1.7	2	2	0	5.7	LQS
Rodríguez, López, Gómez, Rodríguez & Granada (2015)	1	1	0	2	4	MQS
Argudo, Ricardo de la Vega & Ruiz (2015)	2	1	1	2	6	LQS
Gacek (2015)	1	1	2	0	4	HQS
Estevan, Álvarez & Castillo (2016)	1.3	1	2	0	4.3	HQS
Martínez-Romero, Molina & Oriol-Granado (2016)	1.7	1	2	1	5.7	MQS
Abalde & Pino (2016)	1.3	1	2	0	4.3	HQS
Tejero-González, De la Vega-Marcos, Vaquero-Maestre & Ruiz (2016)	2	2	2	0	6	MQS
Gacek (2016)	1	1	2	0	4	LQS
García-Naveira (2017)	1.3	1	2	0	4.3	MQS
Peinado, Cocca, Solano & Blanco (2017)	1.7	2	2	0	5.7	MQS
Segura, Adanis, Barrantes-Brais, Ureña & Sánchez (2018)	1.7	2	1	0	4.7	LQS
García-Naveira (2018)	1.3	1	2	2	6.3	LQS
Molina, Granado & Mendoza Malo (2018)	2	2	2	0	6	LQS
Rubio, Hernández, Sánchez-Iglesias, Cano & Bureo (2018)	2	2	2	0	6	LQS
Malinaukas, Sniras, & Malinaukienes (2018)	1.7	2	2	2	7.7	MQS
Olmedilla, Rubio, Fuster-Parra, Pujals & García-Mas (2018)	2	2	2	2	8	HQS
De Andrade, Gattás & Moura (2019)	1.7	1	0	1.7	4.4	LQS
Salles, Soares, Collet, Milan, Palheta, Mendes, Kós, Nascimento & Carvalho (2019)	2	1	2	0	5	LQS
Chen, Zhang, Yin, Li, Cao, Gutiérrez-García & Guo (2019)	2	2	2	0	6	HQS
Walter, Nikoleizig & Alfermann (2019)	2	1	2	0	5	HQS
Reigal, Vázquez-Diz, Morillo-Baro, Hernández-Mendo & Morales-Sánchez (2019)	2	2	2	0	6	HQS
Martínez-Alvarado, García, Palacios & Rodríguez (2020)	2	2	2	0	6	LQS
Chen, Qiu, Chen, C., Qang, Zhang, & Chai (2020)	1.7	2	2	0	5.7	HQS
Carreres-Ponsoda, Escartí, Jimenez-Olmedo & Cortell-Tormo (2021)	2	2	2	1	7	HQS
Monteiro, D. Monteiro, M. Torregrossa & Travassos (2021)	2	2	2	0	6	MQS
Goraczko, Zurek, Lachowicz, Kujawa & Zurek (2021)	1.3	2	1	0	4.3	LQS
Ramolale, Malete & Ju (2021)	2	2	2	0	6	LQS
Peng & Zhang (2021)	2	2	2	0	6	LQS
Stankovic, Todorovic, Miloševic, Mitrovic & Stojiljkovic (2022)	2	2	2	0	6	MQS
Tang, Liu, Jing, Wang & Yang (2022)	1	1	2	1.3	5.3	LQS
Kwon, Shin & Shin, M. (2022)	2	1	2	0	5	LQS
Rogowska, Tataruch, Niedz´wiecki & Wojciechowska-Maszkowska (2022)	2	1	2	0	5	LQS

## Results of the Synthesis of Articles According to the Analysis Dimensions

1) Descriptive analysis of the formal characteristics of the selected articles:

During the search period (2015–2022), various studies on self-efficacy in high-performance sports were observed, indicating sustained interest in this variable among researchers.

The 34 articles were published in 14 scientific journals, being 67.5 (*n* = 23) from four main journals: *Frontiers in Psychology* (23.5 of the articles, *n* = 8), with an impact factor of 4.2 in JCR and originating from Belgium, specializing in psychology, movement sciences, and sports psychology, prioritizing multidisciplinary studies; *International Journal of Environmental Research and Public Health* (17.6 of the articles, *n* = 6), with an impact factor of 4.614 in JCR and originating from Switzerland, specializing in public, environmental, and occupational health, prioritizing interdisciplinary studies; *Revista de Psicologia del Deporte* (14.7 of the articles, *n* = 6), with an impact factor of 0.7 in JCR and originating from Spain, specializing in sports and exercise psychology, prioritizing multidisciplinary studies; and *Cuadernos de Psicología del Deporte* (11.7 of the articles, *n* = 4), with an impact factor of 0.304 in SJR and originating from Spain, specializing in psychology related to sports sciences. Overall, 71.4 of the analyzed journals (*n* = 9) belonged to the thematic areas of psychology and sports and physical exercise sciences. Regarding publication languages, 62 (*n* = 21) of the articles were in English, and 38 (*n* = 12) in Spanish, excluding publications in Portuguese.

Research on self-efficacy was conducted across different countries, with Spain having the highest number of studies, accounting for 29.4 of the total with 10 investigations. China followed with 11.7 (*n* = 4), and Mexico with 8.8 (*n* = 3). An expansion of self-efficacy studies was observed in various regions, including Europe (52.9%, *n* = 18), Latin America (26.5%, *n* = 10), Asia (14.7%, *n* = 5), and Africa (2.9%, *n* = 1). An exemplary study in this expansion is one conducted with elite athletes from five continents who have experienced spinal cord injuries, correlating self-efficacy with quality of life (Goraczko, 2021).

Investigations were carried out across a total of 30 sports disciplines. Among team sports, soccer (40%, *n* = 13) and basketball (36.7%, *n* = 12) were the most investigated. In individual sports, track and field stood out with 13.3 (*n* = 4), followed by tennis, judo, boxing, and canoeing, each with 8.8 (*n* = 3 articles each). Additionally, studies focused on adapted sports for athletes with disabilities, representing 23.3 of the total (Goraczko, 2021; Tejero-González, 2016). Three studies did not specify the sports discipline of the participants (Peinado et al., 2017; Rodríguez et al., 2015; Tang et al., 2022).

The average annual number of publications between 2015 and 2022 was 4.25 articles, with a standard deviation of 1.57. The year with the highest production was 2018, with six articles, while the year with the lowest production was 2017, with two articles.

2) Methodological approach of the articles and association of variables:

The articles analyzed in this study adopt characteristics of descriptive designs in 33.3 (*n* = 20) and correlational designs in 31.7 (*n* = 19), with 16 of them (41%) presenting a combination of both approaches. Several associated variables (*n* = 36) were identified, demonstrating the breadth of the investigated hypotheses (*Table 3*). Of the total variables, 82.8 are related to psychological variables.

**Table 3:** Variables and Direction of Their Association with Individual and Collective Self-Efficacy

Individual self–efficacy			Collective self–efficacy		
C (+)	C (–)	NC	C (+)	C (–)	NC
Self–confidence in sports competition	Somatic anxiety	Pre–game speeches	–	Team conflict	Individualism
Optimism	Cognitive anxiety	–	–	Role conflict	Chronological age
Competitiveness	Aggressive behavior	–	–	Horizontal collectivism	Maturational stage
Satisfaction with life	Antisocial behavior	–	–	Vertical collectivism / Conformism	–
Social emotional support	Depression	–	–	–	–
Behavior in competition	Occurrence of injury	–	–	–	–
Self–control	–	–	–	–	–
Career goals and planning	–	–	–	–	–
Athletic identity	–	–	–	–	–
Prosocial behavior	–	–	–	–	–
Constancy Mental toughness confidence	–	–	–	–	–
Self–confidence	–	–	–	–	–
Vigor	–	–	–	–	–
Quality of life	–	–	–	–	–
Behavior activation system	–	–	–	–	–
Sporting success	–	–	–	–	–
Observational learning	–	–	–	–	–
Dietary behavior	–	–	–	–	–
Defense performance (soccer)	–	–	–	–	–
Attack performance (soccer)	–	–	–	–	–
Consumption of dietary supplements	–	–	–	–	–

*Note. **C (+) =** Positive Correlation. C (-) = Negative Correlation. NC = No Correlation*

Regarding the association between variables and individual and collective self-efficacy, as presented in Table 3, 61.1 of the articles (*n* = 22) indicated a positive correlation, 27.8 (*n* = 10) showed a negative correlation, and in 11.1 of the studies(*n* = 3), no statistically significant correlation was found between variables such as chronological age, maturation stage, individualism, and self-efficacy, as well as pre-game coach talks (Rivas et al., 2015; Rubio et al., 2018; Salles et al., 2019).

Additionally, 53 of the studies (*n* = 18) established relationships among three or more variables, including self-efficacy (Carreres-Ponsoda et al., 2021; Chen et al., 2019; García-Naveira, 2018; Goraczko et al., 2021; Kwon et al., 2022; Leo, 2015; Molina et al., 2018; Monteiro et al., 2021; Olmedilla et al., 2018; Peng & Zhang, 2021; Ramolale et al., 2021; Reigal et al., 2019; Rivas et al., 2015; Rogowska et al., 2022; Salles et al., 2019; Segura et al., 2018; Tang et al., 2022; Walter et al., 2019).

In studies employing explanatory or experimental designs (*n* = 10), self-efficacy was studied as a dependent variable in eight of them. It was found that self-efficacy was positively influenced by factors such as coach autonomy, socio-emotional support, internal dialogue, mindfulness, activation control training through breathing techniques and biofeedback, personal learning, and social responsibility, as well as by Bandura’s model, verbal persuasion, personal experience, psychophysiological states, and vicarious experience (Carreres-Ponsoda et al., 2021; Malinaukas et al., 2018; Molina et al., 2018; Rodríguez et al., 2015; Tang et al., 2022; Walter et al., 2019). One study found a negative influence of team conflicts on self-efficacy (Leo et al., 2015), and another study found no effect of pre-game coach talks on self-efficacy (Rubio et al., 2018).

In a single study with an experimental design, it was demonstrated that under conditions of different ego threat levels, self-efficacy may not serve as a moderator (Peng & Zhang, 2021).

Among the objectives of the analyzed articles, 11 validations were found, with 4 (36.7%) focusing on interventions and 7 (63.3%) on diagnostic instruments. Validations of intervention programs or models to improve self-efficacy were conducted through designs with control and experimental groups, where instruments were applied before and after the intervention (Carreres-Ponsoda et al., 2021; Malinaukas et al., 2018; Rodríguez et al., 2015; Walter et al., 2019).

Regarding diagnostic instruments, 3 studies (8.8%) presented only psychometric properties, while 4 (11.7%) were part of studies with other objectives to evaluate individual and/or collective self-efficacy based on the domains of sports activity, competitive pressure conditions, and tactical performance. All these studies reported the values of Cronbach’s alpha coefficient (Andrade et al., 2019; Estevan et al., 2016; García-Naveira, 2018; Kwon et al., 2022; Martinez-Alvarado et al., 2020; Martínez-Romero, 2016; Rivas et al., 2015). Three of them consisted of translations and verifications of questionnaires and models applied in new or specific contexts (Andrade et al., 2019; Kwon et al., 2022; Martinez-Alvarado et al., 2020).

3) Results of the self-efficacy measurements, including general and/or specific evaluations:

Out of the 30 studies that addressed self-efficacy assessment, 46.6 applied specific instruments to evaluate specific domains or activities, while 53.3 focused on general self-efficacy. Among these studies, 43.7 used Schwarzer & Jerusalem’s General Self-Efficacy Scale (1995), and 18.7 used Baessler & Schwarzer’s General Self-Efficacy Scale (1996). Self-efficacy was assessed in a general, broad, and stable sense of personal competence in different situations related to optimism, competitiveness, aggressiveness, quality of life after injuries, internal dialogue, quality of life, depression, observational learning, psychological profiles, competitive anxiety, mood states, injury recovery, and the use of dietary supplements (Gacek, 2016; García-Naveira, 2018; Goraczko et al., 2021; Monteiro et al., 2021; Peng & Zhang, 2021; Rogowska et al., 2022; Stanković et al., 2022; Walter et al., 2019). One study conducted both general and specific self-efficacy measurements (Segura et al., 2018).

Regarding microanalyses, 100 of the instruments considered the strength of self-efficacy, 93.3 (*n* = 32) considered its generality, and only 23.33 (*n* = 7) took into account the levels of self-efficacy (Abalde & Pino, 2016; Argudo et al., 2015; Estevan et al., 2016; Leo et al., 2015; Malinaukas et al., 2018; Rivas et al., 2015; Salles et al., 2019). Furthermore, 90 of the studies (*n* = 30) measured self-efficacy individually and 10 (*n* = 4) collectively, regardless of the sport evaluated (Leo et al., 2015; Rivas et al., 2015; Salles et al., 2019).

## Discussion

The theme of self-efficacy in high-performance sports has gained more visibility in sports psychology. There has been an expansion in the number of countries studied, with Spain leading in research output, followed by other European countries, Latin America, and countries from Asia and Africa. Participants from Australia have been involved, with early studies being predominantly English-speaking. A systematic review of self-efficacy measurement instruments found that the majority of articles were from Mexico and published in the Spanish language (Olortegui, 2020). These results align with those found by Martínez-Ramírez et al. (2024), who identified a similar pattern in their systematic review on sports performance and self-efficacy, highlighting the predominance of studies conducted in Latin America and in the Spanish language.

This expansion is in line with the wide range of individual and team sports studied, including adapted sports for athletes with motor disabilities. The use of self-efficacy measures allows athletes to assess their beliefs in coping with adapted sports situations, regardless of the characteristics of the actions involved (Van Raalte et al., 2019). Previous research faced challenges in measuring physical variables for athletes with disabilities due to the lack of standardized tests (Pérez, 2009), but this issue has been overcome. In recent years, there has been international progress in social inclusion, with sports playing a significant role (Tarqui-Silva et al., 2023).

Regarding the sports most chosen for studying self-efficacy in high-performance sports, there have been recent changes. While Balaguer et al. (1995) mentioned that most studies were conducted in individual sports, currently, the team sports soccer and basketball are the most selected. This aligns with Urcino et al. (2019), who found in a review of sports performance evaluation that these two sports were the most commonly evaluated. Soccer, in particular, is highly popular worldwide and attracts considerable interest from researchers. A study focused on football asserted that it is undoubtedly the most mediatized sport and suggested exploring various research topics related to media and science (Meneses & Avalos, 2013).

The most commonly used methodological designs were descriptive-correlational types. There has been diversification in the type of study to assess self-efficacy, as noted by Correa et al. (2022); however, few studies explored and explained the dynamics of self-efficacy in sports activities longitudinally. It would be prudent to support athletes and coaches in their efforts for improvement and to structure work that allows for specific periodization. The importance of quasi-experimental studies in sports psychology lies in the ability to analyze athletes in their natural environments. This approach aligns with one of the main objectives of applied sports psychology: to study athletes during training sessions and competitions within pre-established groups, such as sports teams. By doing so, it becomes possible to observe how independent variables influence dependent ones, allowing for the development of intervention strategies aimed at solving specific issues. Quasi-experimental studies provide a practical framework for identifying the interplay of psychological factors in real-world settings, ultimately bridging the gap between theoretical research and applied practice (Bermúdez-Zea et al., 2024).

Studies showing positive relationships between anxiety, confidence, and self-efficacy persisted, corroborating findings by Balaguer et al. (1995) and Machado et al. (2019). The relationship between performance and self-efficacy was most frequently explored in review articles (Balaguer et al., 1995; Guillén, 2007; Moritz et al., 2000). Moritz et al. (2000) found in a meta-analysis of 45 articles that self-efficacy was more strongly associated with subjective performance measures than with objective ratings, but this did not affect the relationship between the two; Balaguer et al. (1995) reported that many studies found a relationship but could not establish a causal direction.

The most significant finding of the study was the increase in research that established relationships between two or more variables with self-efficacy, affirming the need for integrated understanding and analysis due to the complexity of subjectivity in sports activities (Arruza et al., 1998; Louchbaum et al., 2023).

Exploratory and experimental methodological designs revealed causal relationships, with self-efficacy acting as both a dependent and independent variable. As a dependent variable, it was positively influenced by various practical variables and tools that impacted athletes’ self-efficacy variability, as well as by Bandura’s sources model, as seen in previous reviews (Balaguer et al., 1995; Escarti & García, 1994; Feltz et al., 1992; Yevilao, 2019). Studies on self-efficacy as an independent variable have been limited, with only one case demonstrating that self-efficacy did not serve as a moderator in different ego threat levels.

There has been a decline in experimental studies where self-efficacy explains the movement of another variable, contrary to the findings of Balaguer et al. (1995) in a review of the state of self-efficacy in physical education and sports, where its influence on motor performance was evident in several studies.

Validated interventions provided tools such as internal dialogue, social training, activation control, and teaching personal and social responsibility. These interventions were the only ones within the meta-analysis that were sustained over time through sessions, adapting to longitudinal characteristics of individual or group strategies to solve problems (Olivares et al., 2014). However, addressing how to confront and solve self-efficacy issues continues to be a pending task, as more research focuses on diagnosing the phenomenon of self-efficacy rather than providing solutions for it.

Regarding instrument validation, considerations were made based on Bandura’s (2000) and Feltz’s (1992) arguments about the need to create instruments capable of evaluating specific tasks or domains of the activity. These instruments require a solid conceptual specification of the determinants that govern performance in those domains. For example, individual aspects include technical domains (type of actions for scoring) and tactical domains (attack and counterattack actions) in taekwondo, while collective aspects include situations in attack, defense, offensive transitions, and defensive transitions in football. This is consistent with the findings of Balaguer et al. (1995) and Olortegui (2020), who identified studies using self-efficacy questionnaires created for the specific sport in question.

Despite the above, some studies continue to use general scales. Similar findings were reported by Machado et al. (2019) in a review of self-efficacy in volleyball, who asserted that this remains a limitation and that no study is analyzed based on the psychological demands of the specific activity. This suggests a difficulty in interpreting the construct by authors, since self-efficacy assessment has always specified a task, even at specific levels. Bandura (2000) referred to this in the context of microanalyses in self-efficacy evaluations, and despite considering the dimension of generality, it is formed through the integration of parts or various domains of the task. Studies involving microanalyses in self-efficacy evaluations considered strength and generality in the assessments. Unipolar scales were constructed ranging from zero to maximum belief, using Likert scaling with various response options, as suggested by Bandura (2000), which always implies strength, as found in studies by Balaguer et al. (1995). Levels were less frequently considered, indicating that authors have struggled to implement them within a specific dimension of the facets they represent. These results resemble those obtained by Balaguer et al. (1995). Among 23 English-speaking studies, only five took into account task levels.

The results of this last paragraph suggest a need for a deeper approach. When research in high-performance sports is used to achieve athletes’ performance goals and actions can be implemented to correct issues, the need to involve task levels in self-efficacy assessments becomes more evident. This would help correct and continuously evaluate athletes’ specific beliefs in performance domains as their preparation progresses.

On the other hand, there were also few studies on collective self-efficacy (Leo et al., 2015; Rivas et al., 2015; Salles et al., 2019). Although many studies included team sports, the analysis only considered collective self-efficacy in football and basketball. The measures considered technical, tactical, and skill domains during the game, such as effort, persistence, preparation for overall performance, unity in conflict resolution, communication, and positive attitude. Other studies included group cohesion, leadership, and interpersonal relationships (Black et al., 2019; Judge et al., 2021).

Regarding the analysis of collective self-efficacy with other group-level indicators, there have been advancements. However, compared to individual self-efficacy, there continues to be a limitation in terms of the number of investigations, as previously reported by Machado et al. (2019), or in the objectives of previous reviews (Moritz et al., 2000; Olortegui, 2020; Yevilao, 2019).

## Conclusion

Self-efficacy in high-performance sports continues to be of interest to researchers. There has been an expansion in research to include Europe, Asia, and Africa, and numerous sports, with football and basketball being the most studied, along with a notable inclusion of adapted sports. The results show that a large number of studies are descriptive-correlational, with a positive increase in research that establishes relationships between two or more variables with self-efficacy, highlighting the complexity of the analyses. Experimental studies, in terms of causal relationships, showed limited results in which self-efficacy explains the movement of another variable. However, as a dependent variable, some studies demonstrated that self-efficacy was positively influenced by variables that impacted athletes’ self-efficacy variability, and by Bandura’s sources model. This is consistent with the validated interventions, which incorporated variables and tools that explain the increase in self-efficacy. Validated interventions were the only ones within the meta-analysis that included longitudinal studies, which constituted a limitation in self-efficacy research. Many studies still rely on cross-sectional designs. For microanalysis in self-efficacy evaluation instruments, considerations were made regarding strength and generality, but the inclusion of levels in the domains was found to be limited. Considering the dimension of time could offer the opportunity for precise knowledge of the activity and its development in the preparation stages. Self-efficacy in these performances could be analyzed as the preparation progresses. This scientific gap opens the way for new research. Regarding the evaluation of self-efficacy individually and collectively, the former was more widely studied, even for participants in team sports. Another aspect to consider is the formulation of new contributions to collective self-efficacy, considering intrinsic indicators and variables related to dynamics in team sports.

## Limitations

It is possible that there are other studies of self-efficacy in high-performance sports that are found in other databases and that provide new considerations. These analyses are limited to these indicators that do not preclude the possibility of other findings. One of the primary limitations of this study was the accessibility of databases. While the selected databases were chosen for their international visibility and recognition, access to certain resources proved to be a constraint. It is known that some databases include content from others; for example, PubMed offers full-text documents from many biomedical journals, including those indexed in Medline, as well as articles from other fields such as physics, chemistry, and astrophysics, many of which are available for free.

Another important limitation lies in the geographic and linguistic disparities within the databases. Renowned databases such as Scopus and Web of Science (WoS) exhibit regional limitations that affect accessibility and representation. Additionally, the ecosystem of scientific articles reflects a division between commercial publishers, who historically published in print and now sell digital access to journals, and non-commercial publishers, which are typically institutions, scientific societies, or non-profit organizations. These factors may have influenced the comprehensiveness of the review by limiting access to certain regional or open-access studies.

The findings of this study provide valuable insights for practitioners and researchers in high-performance sports. Coaches and sports psychologists can leverage validated interventions that incorporate Bandura’s self-efficacy sources to enhance athletes’ confidence and performance. These interventions, particularly when applied longitudinally, have been shown to positively influence self-efficacy and its variability over time. Furthermore, considering self-efficacy as a dynamic variable throughout different preparation stages offers a practical framework for tailoring interventions to athletes’ needs during training and competition phases. The limited exploration of collective self-efficacy in team sports highlights the need to address team dynamics and intrinsic indicators, which could foster stronger team cohesion and collective performance. Addressing the current over-reliance on cross-sectional designs, future research should aim to incorporate experimental and longitudinal studies to establish causal relationships and provide a deeper understanding of how self-efficacy evolves over time. This could ultimately aid in designing more effective training programs that improve both individual and team outcomes in high-performance sports contexts.
